# Tracking cancer lesions on surgical samples of gastric cancer by artificial intelligent algorithms

**DOI:** 10.7150/jca.63879

**Published:** 2021-09-03

**Authors:** Ruixin Yang, Chao Yan, Sheng Lu, Jun Li, Jun Ji, Ranlin Yan, Fei Yuan, Zhenggang Zhu, Yingyan Yu

**Affiliations:** 1 Department of General Surgery of Ruijin Hospital, Shanghai Institute of Digestive Surgery, and Shanghai Key Laboratory for Gastric Neoplasms, Shanghai Jiao Tong University School of Medicine, 200025, Shanghai, China.; 2 Department of Pathology of Ruijin Hospital, Shanghai Jiao Tong University School of Medicine, 200025, Shanghai, China.

**Keywords:** Artificial Intelligence, Object Detection, Semantic Segmentation, Gastric Cancer, Macroscopic Images

## Abstract

To quickly locate cancer lesions, especially suspected metastatic lesions after gastrectomy, AI algorithms of object detection and semantic segmentation were established. A total of 509 macroscopic images from 381 patients were collected. The RFB-SSD object detection algorithm and ResNet50-PSPNet semantic segmentation algorithm were used. Another 57 macroscopic images from 48 patients were collected for prospective verification. We used mAP as the metrics of object detection. The best mAP was 95.90% with an average of 89.89% in the test set. The mAP reached 92.60% in validation set. We used mIoU for evaluation of semantic segmentation. The best mIoU was 80.97% with an average of 79.26% in the test set. In addition, 81 out of 92 (88.04%) gastric specimens were accurately predicted for the cancer lesion located at the serosa by ResNet50-PSPNet semantic segmentation model. The positive rate and accuracy of AI prediction were different based on cancer invasive depth. The metastatic lymph nodes were predicted in 24 cases by semantic segmentation model. Among them, 18 cases were confirmed by pathology. The predictive accuracy was 75.00%. Our well-trained AI algorithms effectively identified the subtle features of gastric cancer in resected specimens that may be missed by naked eyes. Taken together, AI algorithms could assist clinical doctors quickly locating cancer lesions and improve their work efficiency.

## Introduction

Gastric cancer is one of the most common gastrointestinal cancers with the fifth incidence and the second mortality worldwide 1. The rapid and accurate diagnosis of gastric cancer will improve the treatment outcome of patients. Gastrectomy is a key therapeutic measure for gastric cancer. After gastrectomy, pathologists will examine and report the cancer histology and possible metastatic status. This procedure takes a lot of time, especially when the cancer is at its early stage. Therefore, quickly locating the cancer lesions, especially suspected metastatic lesions will improve the work efficiency. In this study, artificial intelligence (AI) algorithms of object detection and semantic segmentation were used to identify gastric cancer in surgical specimens.

AI progresses quickly in the field of medical images 2. Convolutional neural network (CNN) is a deep learning network for computer vision analysis 3. It is proper for analyzing medical images including image classification, object detection and semantic segmentation. Image classification using CNN for gastric mucosal lesions has achieved progression in our previous study 4. Using the excellent ability of CNN for feature extraction, more robust algorithms of object detection and semantic segmentation are being developed 5. There are a variety of CNN models with different precision and training speed including VGG, Inception, ResNet, MobileNet, Xception, EfficientNet, and so on 6-9.

To identify the cancer lesions and suspected metastatic lymph nodes accurately, we constructed the object detection and semantic segmentation algorithms with CNN backbones. The AI algorithms could accurately recognize the gastric cancer lesions in macroscopic images, and also predict metastatic lesions in surrounding lymph nodes. To our knowledge, this is the first report that AI technology could be used for assisting cancer lesion recognition in post-operative specimens.

## Methods

### Model construction and image labeling

All macroscopic images of stomach were obtained after gastrectomy as JPG format. The inclusion criteria was the specimens come from surgical resection of confirmed gastric cancer. The exclusion criteria included low resolution images, unclear location or obvious bleeding. A total of 509 macroscopic images from 381 patients of gastric cancer were included in the object detection and semantic segmentation analysis. Among them, 460 out of 509 (90%) images from 347 patients were used as the training set, and 49 out of 509 (10%) images from 34 patients as test set. All images were obtained from the Department of General Surgery, Ruijin Hospital of Shanghai Jiaotong University School of Medicine. This study was approved by the Research Ethics Committee of Shanghai Ruijin hospital. The written informed consents were signed by all patients.

Macroscopic images were labeled by surgical experts (YC and YR) with LabelImg (an open-source platform for object detection image annotations; https://github.com/tzutalin/labelImg), and Labelme (an open-source platform for semantic segmentation image annotations; https://github.com/wkentaro/labelme). The key steps included inputting of macroscopic image, manual labeling and image outputting. In object detection analysis, the macroscopic images were manually labeled with priori boxes, and then saved with Human-readable Extensible Markup Language (XML) format. In semantic segmentation analysis, macroscopic images were manually labeled and saved with boundary and JavaScript Object Notation (JSON) format. The subsequent analysis would extract labeling information from JSON files to transfer original images into pixels images with red lesion (255, 0, 0) and black background (0, 0, 0).

### Prospective validation set

The prospective validation set was an independent set for evaluating model performance to ensure the generalization ability. All images were saved in JPG format. The exclusion criteria were the same as that of the training set. Finally, 57 images from 48 patients were included. Images in the prospective validation set did not overlap with that in the training set.

### The serosal appearance analysis

To analyze the involving status of gastric serosa, we collected 92 resected stomach specimens from advanced gastric cancer. These images were used for predicting serosal invasion at the status of no incision of stomach.

### K-fold cross validation

K-fold cross validation was used according to previous reports for getting better training efficiency when the primary data was limited 10, 11. After K times training, the best model weight was defined according to the evaluation in each fold using the test set.

### Computational requirements and algorithms

The study was performed using the hardware Intel Core i7-10750H CPU, 16G RAM, NVIDIA GeForce RTX 2060 and the 6G VRAM.VOC2007 (Visual Object Classes2007) was used for transfer learning images 12, 13. By preliminary tests on 12 object detection models (MobileNet-SSD, VGG16-SSD, RFB-SSD, YOLO-V3, YOLO-V4, YOLO-V4-tiny, RetinaNet, M2det, CenterNet, EfficientDetD0 and D1, and Faster RCNN) 8, 9, 14-17 and 9 semantic segmentation models (MobileNet-Unet, VGG16-Unet, MobileNet-PSPNet, ResNet50-PSPNet, CENet, FCN, CFPNet, DCUNet and ICNet) 18-20, the RFB-SSD object detection and PSPNet semantic segmentation revealed the best performance, and were used in this study. All computation was run on the Google's Tensorflow and Keras deep learning framework based on python language 21.

### Construction of RFB-SSD object detection model

The Receptive Field Block-Single Shot MultiBox Detector (RFB-SSD) model was the integration of RFB module with VGG16, and it increased the ability of feature extraction and object detection^22^. To obtain the best performance for detecting gastric cancer in macroscopic images, RFB-SSD model was dynamically trained by 10-fold cross validation with the maximum epochs as 100. In the training process, Adam was used to optimize convergence speed. The MultiboxLoss was used to reduce the imbalance of the positive and negative samples^14^. The input size of training and evaluating images were 300×300×3 pixels. The evaluation parameters of object detection include mean average precision (mAP), log-average miss-rate (LAMR), precision, recall and F1.

### Construction of ResNet50-PSPNet semantic segmentation model

The pyramid pooling module was introduced into PSPNet semantic segmentation with multiple convolutional kernels of different size for construction of pooling feature pyramids 23. The key steps include ResNet50 preliminary feature extraction and PSPNet enhanced feature extraction. To obtain the best performance, ResNet50-PSPNet model was dynamically trained by 10-fold cross validation with the maximum epochs as 100. In the training process, Adam was used to optimize semantic segmentation. The dice loss was used as the loss function to reduce the imbalance of the positive and negative samples 24. The input size of the training and evaluating images were 273×273×3 pixels. The evaluation parameters of semantic segmentation include intersection over union per class (class IoU) and mean IoU (mIoU).

### Statistical analysis

In the object detection algorithm, the evaluation parameters include mAP, LAMR, precision, recall and F1. In the semantic segmentation algorithm, the evaluation parameters include IoU and mIoU. The difference of different algorithms was compared by Wilcoxon paired signed-rank test with *p<0.05* as statistical significance. All tests were bilateral. The Prism (8.0, GraphPad) was used for statistical analysis and plotting.

## Results

### The performance of object detection algorithm

**Figure 1:**
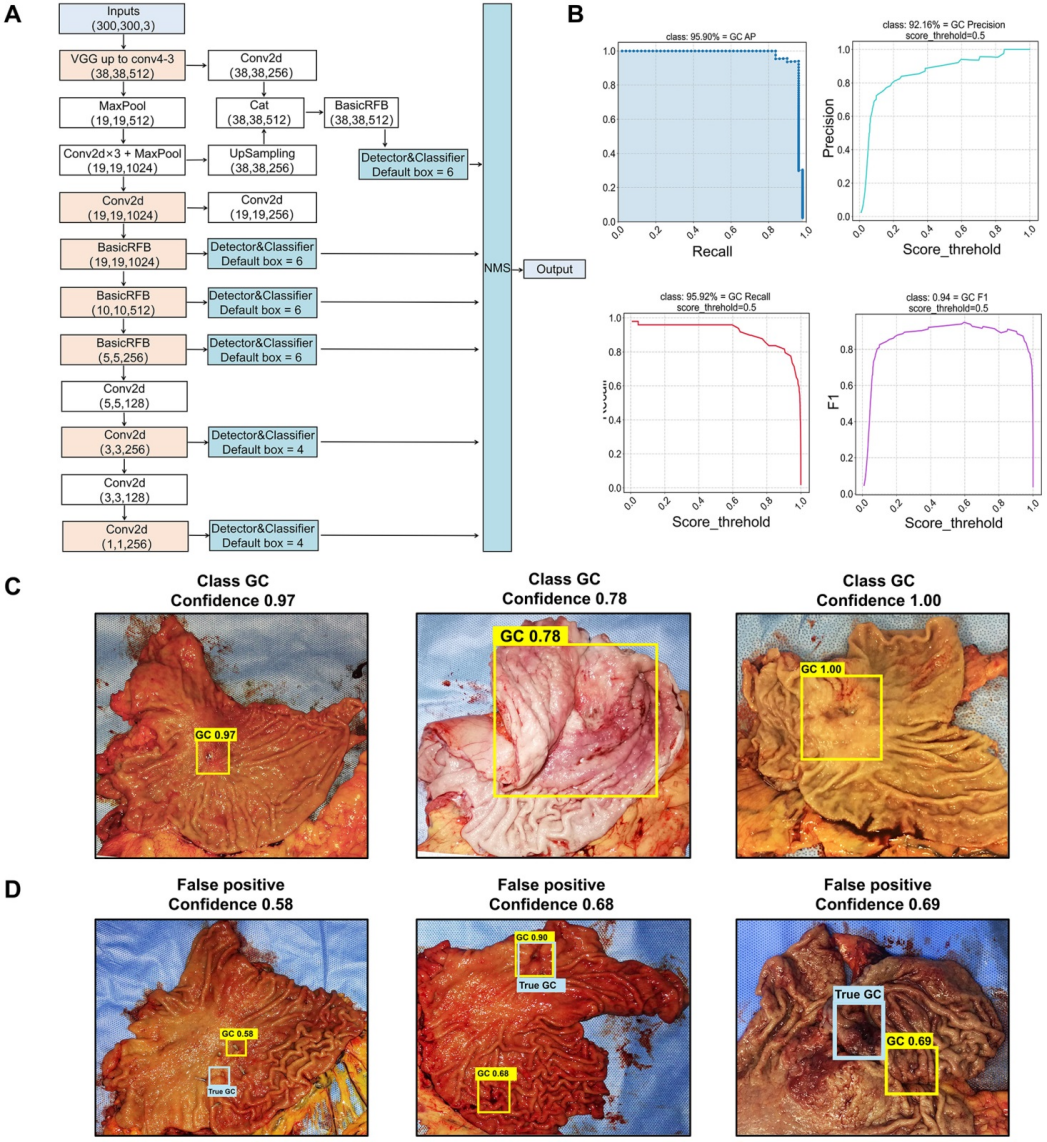
** Identification of cancer lesions by the RFB-SSD object detection algorithm. A.** The structure of RFB-SSD model. **B.** The area under the curve of precision and recall (mAP) reached 95.90% (left up). At 0.5 threshold value, the precision score reached 92.16% (right up), the recall score 95.92% (left down) and the F1 score 0.94 (right down). **C.** True-positive prediction results. All cancer lesions were correctly predicted with high confidence shown on the top of yellow boxes. The predictive confidence of true-positive cases was over 0.7 (0.97±0.07). **D.** False-positive predictive results. The blue labels indicated the true-positive lesions, and the yellow labels represented the false-positive lesions with low confidence (0.65±0.05).

Among the 12 selected object detection algorithms, RFB-SSD algorithm **(Figure 1A)** revealed the highest mAP of 96.36%, and the lowest LAMR of 0.000001 with 90% precision, 90% recall and F1 0.9 **(Table 1)**. So, RFB-SSD was used for the subsequent analysis. Based on the 10-fold cross validation in the training set **(Table S1)**, the mean mAP reached 89.89% (95% CI, 87.49%-92.29%), and the LAMR reached 0.16 (95% CI, 0.12-0.20). At the 0.5 score threshold, the precision was 88.21% (95% CI, 85.92% -90.49%), the recall was 88.37% (95% CI, 85.79%-90.95%), and the F1 was 0.89 (95% CI, 0.86-0.91). In the test set, the best prediction accuracy reached 91.84% with the mAP of 95.90% **(Figure 1B,** left up**)**. The LAMR reached 0.07 at the fold four steps. At the 0.5 score threshold, the precision was 92.16% (**Figure 1B,** right up), the recall was 95.92% (**Figure 1B,** left down), and the F1 was 0.94 (**Figure 1B,** right down).

In the test set, the prediction accuracy for gastric cancer was 91.84% and the false negative rate (FNR) was 8.16%. For the true-positive lesions, the confidence and classification result presented on the top of the yellow boxes were of a high confidence (0.97±0.07)** (Figure 1C)**. Three lesions fell into the false-positive category with a low confidence (0.65±0.05) **(Figure 1D)**. The confidence between the true-positive and false-positive groups were significantly different (*p<*0.0001). We further analyzed the reason of false-positive cases and found that the predictive box of one case was overlapped with the rugae of the greater curvature of the stomach, which mimicked ulcerative lesion **(Figure 1D,** left**)**. In another case, the predictive box overlapped with a surgical metal clip, which interfered with the AI analysis **(Figure 1D,** mid**)**. The last false-positive case was the gastric stump with distorted anatomical structure, which was rarely learnt by AI during the training procedure **(Figure 1D,** right**)**. In addition, the AI failed for predicting lesions in two cases, one was a diffuse type lesion (type Borrmann IV) lacking obvious lesion boundary and the other was from the gastric stump with distorted anatomical structure, which was rarely learnt by AI during the training procedure. The results showed that the RFB-SSD object detection algorithm performed well in positioning cancer lesions in resected specimens. This algorithm revealed fast speed and high accuracy.

### The performance of semantic segmentation algorithm

**Figure 2:**
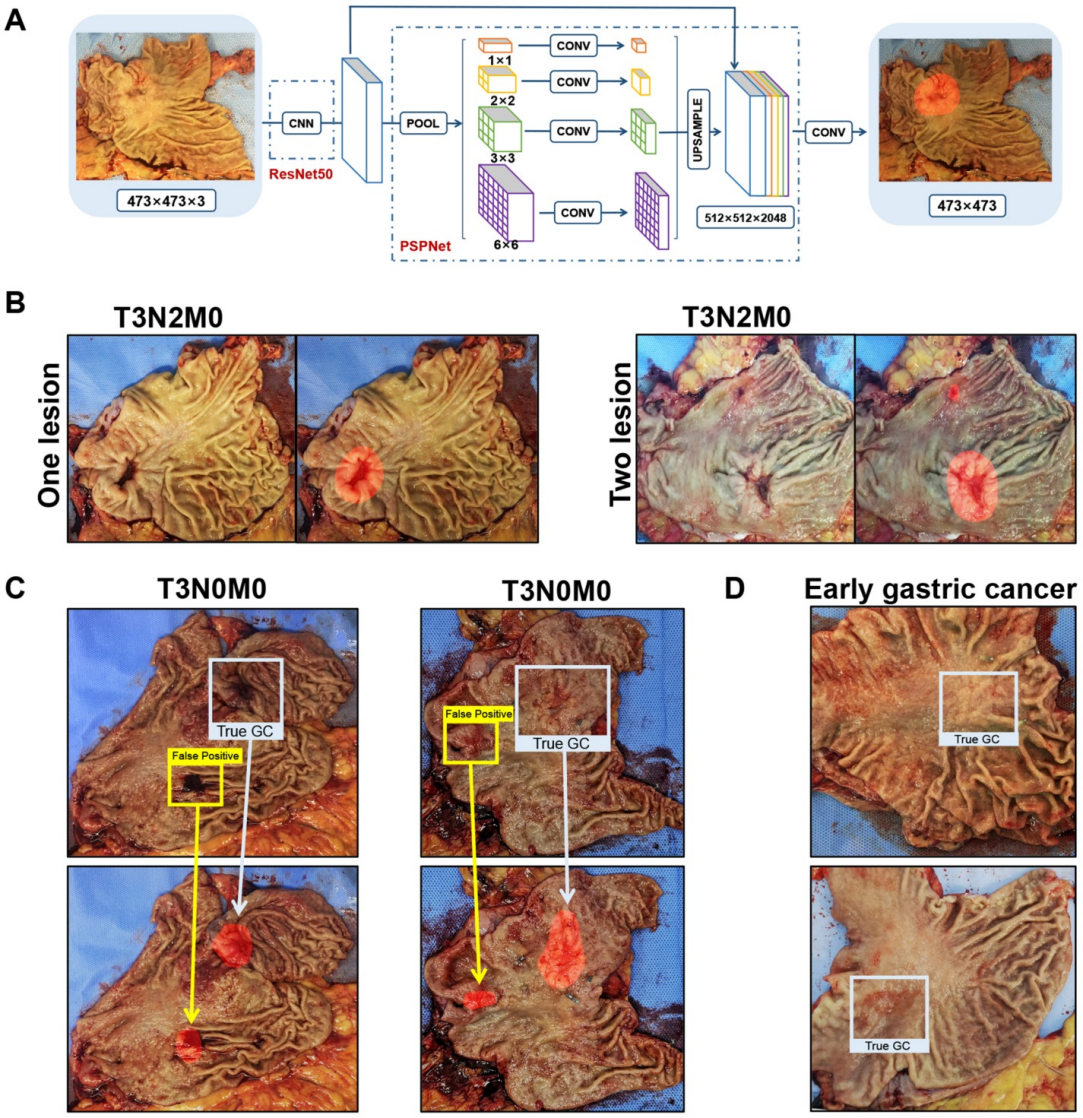
** Identification of cancer lesions by the ResNet50-PSPNet semantic segmentation algorithm. A.** The network of ResNet50-PSPNet structure. ResNet50 was used as CNN backbone for feature extraction, and PSPNet for semantic segmentation. In the output picture, the predictive region was highlighted as red. **B.** The true-positive cases were accurately predicted and highlighted as red. **C.** When gastric mucosa was attached by blood clot (left) or metal clip (right), the computer was confused with false-positive prediction. The red highlighted regions showed the predicted results. The blue boxes labeled true-positive regions, and the yellow boxes labeled the false-positive regions. **D.** Both failed predictions were early gastric cancers.

Among the nine selected semantic segmentation algorithms, the ResNet50-PSPNet revealed the highest mIoU **(Table 2)**. So, the ResNet50-PSPNet was used for the subsequent analysis **(Figure 2A)**. After 10-fold cross validation **(Table S2)**, the IoU reached 62.36% (95% CI, 60.75 %-63.97%), and the mIoU reached 79.26% (95% CI, 78.34%-80.18%). In the test set, the best IoU and mIoU reached 65.38% and 80.97%, respectively. The predictive accuracy reached 93.87% **(Figure 2B),** and the FNR was 6.13%. We noticed that the computer might be confused and resulted in false-positive prediction if the mucosa is attached by blood clot or surgical metal clip **(Figure 2C)**. The predicting lesions failed in two cases. Both of them were early gastric cancer with superficial depression type, which was rarely learnt by the AI during the training procedure** (Figure 2D)**. In this study, by comparing nine competitive semantic segmentation algorithms, the ResNet50-PSPNet semantic segmentation algorithm performed best with the highest mIoU for positioning cancer lesions.

### The performance of both algorithms in prospective validation set

**Figure 3:**
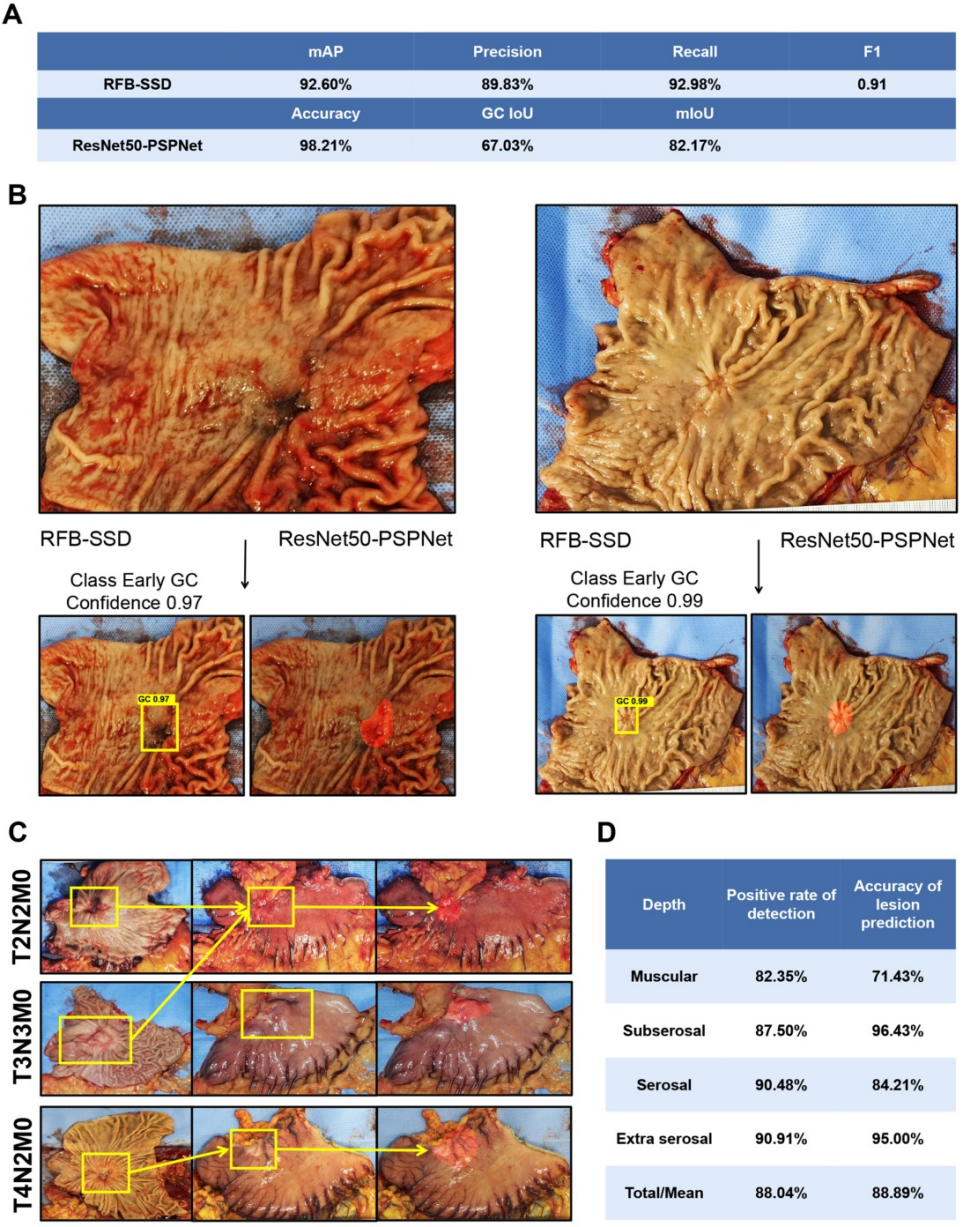
** Evaluation of predictive ability by both algorithms on the validation set and the serosal invasion set. A.** The performance of RFB-SSD and ResNet50-PSPNet algorithms in the validation set. **B.** The predictive result of early gastric cancer by the RFB-SSD algorithm and ResNet50-PSPNet algorithm. **C.** The predictive result of serosa invasion by the ResNet50-PSPNet algorithm analysis. **D.** The positive rate and accuracy of AI prediction based on cancer invasive depth.

A total of 57 images from 48 patients were included in the prospective validation set. Using the RFB-SSD object detection algorithm, all lesions were detected with 100.00% accuracy and mAP 92.60%. At the 0.5 threshold, the precision, recall, and F1 were 89.83%, 92.98% and 0.91 respectively **(Figure 3A)**. By the ResNet50-PSPNet semantic segmentation algorithm, the accuracy of locating cancer lesions reached 98.21% with FNR 1.79%. The IoU and mIoU were 67.03% and 82.17% respectively **(Figure 3A)**. Both algorithms revealed good generalization ability for positioning cancer lesions. The time using both RFB-SSD and ResNet50-PSPNet algorithms for the 57 images was only 8s and 7s respectively.

We compared the performance of detecting and segmenting lesions by the RFB-SSD and ResNet50-PSPNet models with clinical doctors (**Table 3**). The diagnostic mAP of RFB-SSD was significantly higher than that of the doctors. The diagnostic mIoU of ResNet50-PSPNet was also significantly higher than that of the doctors. Furthermore, the time for detecting and labeling these 57 images were just several seconds by both RFB-SSD and ResNet50-PSPNet algorithms. The labeling time for these 57 images was over 5 minutes for RFB-SSD analysis, and was 18 minutes for ResNet50-PSPNet analysis by the doctors.

We separately analyzed the predictive ability of both algorithms for early gastric cancer (16 images from 14 patients) and advanced gastric cancer **(Figure 3B)**. For early gastric cancer set, the accuracy was 93.75% and the FNR was 6.25% with an mAP of 84.17%. At 0.5 threshold, the precision, recall, and F1 were 87.50%, 87.50%, and 0.88, respectively by the RFB-SSD algorithm. Using the ResNet50-PSPNet semantic segmentation algorithm, the predicted accuracy was 81.25% with IoU and mIoU 37.52% and 67.99% respectively. For the advanced gastric cancer set, the mAP was 95.70%. At the 0.5 threshold, the precision, recall, and F1 were 91.40%, 95.51%, and 0.93 respectively by the RFB-SSD algorithm. By the ResNet50-PSPNet semantic segmentation algorithm, the IoU and mIoU were 67.11% and 81.91% respectively. Therefore, both RFB-SSD and ResNet50-PSPNet algorithms revealed good generalization ability and clinical application potential.

### The performance of semantic segmentation for serosal invasion

A total of 92 images of resected gastric specimens were enrolled into serosal invasion analysis by ResNet50-PSPNet algorithm. According to the pathological records, the invasion of muscular layer, subserosal layer, serosal layer, and extra serosa was 17 cases, 32 cases, 21 cases, and 22 cases, respectively. By the ResNet50-PSPNet analysis, the cancer location at serosa of 81 lesions were predicted accurately, and highlighted as red **(Figure 3C)**. The predictive rate was 88.04%. The accuracy of positive cases was 88.89% and the FNR were 11.11%. The positive rate and accuracy of AI prediction were different based on their invasive depth **(Figure 3D)**. Therefore, the ResNet50-PSPNet algorithm revealed the high accuracy for predicting serosal invasion.

### Performance of semantic segmentation for predicting lymph nodes metastasis

**Figure 4:**
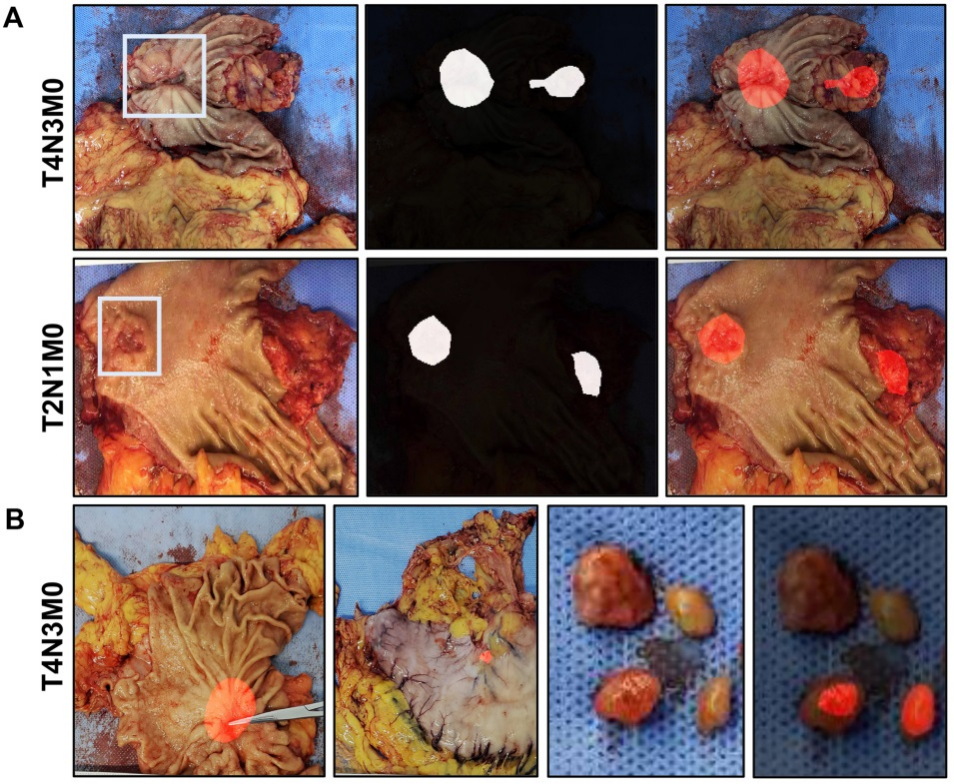
** The predictive results of lymph node metastasis by ResNet50-PSPNet algorithm. A.** A primary cancer region and a suspected metastatic area of surrounding lymph nodes were highlighted as red. **B.** The predictive results of primary cancer lesion, the serosa invasion, and the resected lymph nodes in one case were presented. The cancer lesion and the suspected lymph nodes metastasis were highlighted as red.

A total of 556 images from 429 patients (training set plus validation set) were enrolled for prediction of lymph node metastasis. The lymph nodes metastasis was reported in 253 out of 429 cases (58.97%) by pathology. By the ResNet50-PSPNet analysis, lymph node metastasis was predicted in 24 cases (5.59%) **(Figure 4A)**. Among them, 18 cases were confirmed by pathology (75%). The predictive results of primary cancer lesion, the serosa invasion, and the resected lymph nodes in one case were presented in **Figure 4B**. This result suggested that the ResNet50-PSPNet algorithm revealed limited predicting ability in cancer metastasis to surrounding lymph nodes.

## Discussion

Most medical image analysis by AI was focused on disease classification 25. Along with the development of AI, several new algorithms revealed powerful functions 5. For instance, object detection and semantic segmentation algorithms are gradually applied in medical image research including endoscopic images, computed-tomography (CT) images and pathological images 25-33. However, there is a lack of AI analysis for medical images from macroscopic specimens. Actually, there are a lot of information in macroscopic specimens, such as identification of lesion location (especially for early stage lesion), the cancer classification, surrounding lymph nodes metastasis, and so on. Since our group photographed resected specimens in routine works, we initiated the current study. To our knowledge, this is the first report that AI technology can be used for macroscopic specimen analysis in gastric cancer.

Recently, scientists tried to integrate the traditional CNN algorithms with object detection and semantic segmentation. The purpose of object detection is to make sure whether there are objects in predefined images. Hirasawa and coworkers utilized the SSD object detection model to accurately recognize early gastric cancer in endoscopic images. The performance of AI detection had higher sensitivity than that of endoscopic physicians 26, 34. Semantic segmentation is another algorithm in which computer segments images based on the pixels presented in the images. Luo and coworkers utilized DeepLab v3+ semantic segmentation algorithm to precisely segment upper gastrointestinal malignancies in endoscopic images 30. However, there is no report on using AI technology for recognizing cancer lesions in macroscopic specimens. To find the proper models of the above two algorithms, we screened the performance of 12 object detection models and nine semantic segmentation models. Based on their performance, we proposed that the RFB-SSD object detection model and the ResNet50-PSPNet semantic segmentation model are well performed for macroscopic specimen analysis of gastric cancer. Up to date, there is no report utilizing the RFB-SSD object detection model for analyzing medical images. However, PSPNet semantic segmentation model had been used to analyze CT images previously 20. These two models have been widely used in general images analysis of daily life 22, 35-37.

In the current study, the object detection algorithm provides an accurate recognition of cancer lesions with a high confidence value. For the true-positive cases, all confidence values are 0.7 or above, while the confidence values of false positive cases are lower than 0.7. The semantic segmentation algorithm provides an automatic segmentation of cancer lesions. Because the large amount of images in our study come from advanced gastric cancer, both RFB-SSD object detection and ResNet50-PSPNet semantic segmentation models performed well in specimens of advanced gastric cancer relative to early gastric cancer. We assumed that we could improve the performance of the models if we collect more images from early gastric cancer and train these models.

In addition, we found that the ResNet50-PSPNet semantic segmentation model can not only recognize and segment primary cancer lesions, but also predict cancer involvement in serosa. This function will guide doctors to avoid damaging cancer lesions when they open the stomach. As to some positively predicted cases with muscular layer invasion, there might be two possible reasons. One is the missed diagnosis of invasive depth by histology. Another is that the invasion of muscular layer by cancer stimulates the cancer-specific fibrosis of serosa. The subtle texture change will be identified by AI. Based on this ability, the ResNet50-PSPNet semantic segmentation model could predict lymph nodes metastasis in a portion of cases. Although the positive predictive rate of lymph nodes is limited, this function of the ResNet50-PSPNet semantic segmentation model is worth for further development.

We further compared the performances of the two models with clinical doctors on the parameters of mAP, precision, recall, F1, accuracy, FNR, mIoU and using time. We found that both models performed better than the doctors. Therefore, AI aided- positioning cancer lesions could improve working efficiency. Since the AI models can not only locate cancer lesions, but also predict serosa involvement as well as lymph nodes metastasis in some cases, AI will have great potential in clinical application. However, both the RFB-SSD and ResNet50-PSPNet models used in the present study are supervised algorithms in AI area. One of the disadvantages of supervised algorithms is the manual annotation, which is expensive and time-consuming. At present, unsupervised AI algorithms are gradually developed, which could learn high-quality features without the use of manual labels. Unsupervised learning algorithms have not been widely applied in medical images. Recently, Sari and co-workers have applied unsupervised feature extraction algorithms for colon cancer pathological image classification 38. We believe that along with the utility of unsupervised algorithms, it not only reduces the laborious manual annotation work, but also finds out subtle texture features that may miss by naked eye.

Our study had some limitations. The performance of both models for detecting early gastric cancer lesions was not satisfactory. Because the morphological difference between early gastric cancer with surrounding normal mucosa is not significant, how to improve the performance of AI algorithms for recognizing early gastric cancer is the research direction in the future. We will collect more images from early gastric cancer to train the AI models in our future study. Another limitation is that all images are static. We could not predict the cancer lesions in living status. We will try to develop and integrate the models into the computer of laparoscopic devices for real-time recognition of cancer invasion or lymph nodes metastasis. In addition, the current study is a single center research. A multi-center study to validate the performances of the RFB-SSD and ResNet50-PSPNet models is warranted.

## Conclusion

**Figure 5:**
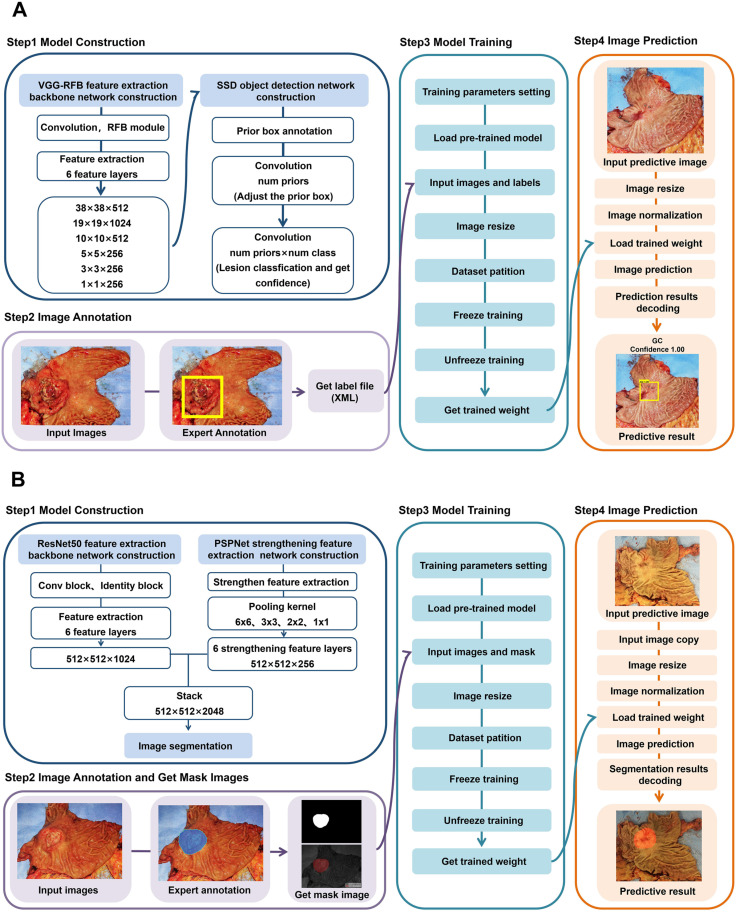
** The flow diagrams of RFB-SSD object detection and ResNet50-PSPNet semantic segmentation algorithms. A**. The step by step flow diagram of RFB-SSD object detection analysis. **B.** The step by step flow diagram of ResNet50-PSPNet semantic segmentation analysis.

This study reported a RFB-SSD object detection model **(Figure 5A)** and a ResNet50-PSPNet semantic segmentation model **(Figure 5B)** for macroscopic specimens analysis of gastric cancer. Both algorithms effectively identified the subtle features of cancer lesions that may be missed by naked eyes. The AI algorithms could assist doctors quickly locating cancer lesions and improve work efficiency.

## Supplementary Material

Supplementary tables.

## Figures and Tables

**Table 1:** The performance of several object detection models

Models	Feature extraction network	Object detection	mAP	LAMR	F1	Precision	Recall
MobileNet-SSD	MobileNet	SSD	85.73%	2.6×10^-1^	0.76	93.55%	64.44%
VGG16-SSD	VGG16	SSD	77.99%	3.5×10^-1^	0.74	77.78%	70.00%
YOLO-V3	Darknet53	YOLO-V3	74.90%	3.1×10^-1^	0.75	100.00%	60.00%
YOLO-V4	CSPDarknet53	YOLO-V4	85.36%	2.4×10^-1^	0.88	97.22%	79.55%
YOLO-V4-tiny	CSPDarknet53	YOLO-V4-tiny	81.00%	2.9×10^-1^	0.75	100.00%	60.00%
RFB-SSD	VGG-RFB	SSD	96.36%	2.9×10^-6^	0.90	90.00%	90.00%
Retinanet	ResNet50	Retinanet	89.28%	2.9×10^-3^	0.86	81.82%	90.00%
M2det	VGG16	M2det	71.54%	3.0×10^-1^	0.82	100.00%	70.00%
CenterNet	ResNet50	CenterNet	73.47%	3.6×10^-1^	0.63	95.45%	46.67%
EfficientDet D0	EfficientNetD0	EfficientDet	94.08%	1.2×10^-1^	0.85	90.00%	80.00%
EfficientDet D1	EfficientNetD1	EfficientDet	94.34%	1.2×10^-1^	0.86	90.24%	82.22%
Faster RCNN	ResNet50	Faster RCNN	88.18%	2.1×10^-1^	0.72	60.00%	90.00%

**Table 2:** The performance of several semantic segmentation models

Models	Feature extraction network	Semantic segmentation	mIoU
MobileNet-PSPNet	MobileNet-v2	PSPNet	74.12%
ResNet50-PSPNet	ResNet50	PSPNet	79.26%
MobileNet-UNet	MobileNet	UNet	74.01%
VGG16-UNet	VGG16	UNet	73.33%
CENet	ResNet34	CENet	44.86%
FCN	VGG16	FCN	22.88%
CFPNet	CNN	CFPNet	55.32%
DCUNet	CNN	DCUNet	57.15%
ICNet	CNN	ICNet	48.86%

**Table 3:** The performance comparison between doctors and AI in validation set

	RFB-SSD							ResNet50-PSPNet	
	mAP	Precision	Recall	F1	Accuracy	FNR	Time	mIoU	Time
Doctor 1	62.34%	68.85%	75.00%	0.72	94.64%	5.36%	12min 30s	76.67%	18min 12s
Doctor 2	47.62%	63.16%	64.29%	0.64	91.07%	8.93%	15min 40s	76.11%	20min 5s
Doctor 3	57.14%	75.00%	75.00%	0.75	96.40%	3.60%	6min 41s	81.63%	23min 48s
AI model	92.60%	89.83%	92.98%	0.91	100.00%	0.00%	8s	82.17%	7s
