# Supplementary material for: Tracking cancer lesions on surgical samples of gastric cancer by artificial intelligent algorithms
Source: J Cancer. 2021 Sep 3;12(21):6473–83. doi: 10.7150/jca.63879 (PMC8489126; doi:10.7150/jca.63879)
Supplement: Supplementary file 1 — Supplementary tables. [file jcav12p6473s1.pdf]

Table S1. The 10-fold cross test results of RFB-SSD

	mAP(%)	Precision(%)	Recall(%)	F1	LAMR
Fold1	86.17	89.58	87.76	0.89	0.21
Fold2	89.84	88.00	89.80	0.89	0.16
Fold3	92.31	91.84	91.84	0.92	0.12
**Fold4**	**95.90**	**92.16**	**95.92**	**0.94**	**0.07**
Fold5	87.67	85.42	83.67	0.85	0.20
Fold6	87.14	84.31	87.76	0.86	0.19
Fold7	91.32	86.00	87.76	0.87	0.14
Fold8	90.13	89.58	87.76	0.89	0.15
Fold9	93.08	91.49	87.76	0.90	0.13
Fold10	85.36	83.67	83.67	0.84	0.25
Mean	89.89	88.21	88.37	0.89	0.16
Lower 95% CI	87.49	85.92	85.79	0.86	0.12
Upper 95% CI	92.29	90.49	90.95	0.91	0.20

Table S2. The 10-fold cross test results of ResNet50-PSPNet

	GC IoU	Background IoU	mIoU
Fold1	62.44	96.21	79.32
Fold2	63.24	96.19	79.72
Fold3	58.81	95.51	77.16
Fold4	63.37	96.4	79.89
Fold5	63.5	96.21	79.85
Fold6	60.06	95.98	78.02
Fold7	59.33	95.74	77.54
Fold8	62.58	96.37	79.48
Fold9	64.89	96.45	80.67
**Fold10**	**65.38**	**96.56**	**80.97**
Mean	62.36	96.16	79.26
Lower 95% CI	60.75	95.93	78.34
Upper 95% CI	63.97	96.40	80.18

Table S3.The clinical information of gastric cancer used in this study

Characteristics	Testing dataset	Prospective validation dataset
Male/female, No. (%)	23/11(67.65%/32.35%)	40/8(83.33%/16.67%)
Age, median, (range), years	62.56(11.87)	67.31(10.01)
Tumor size, median, (range), mm	3.87(2.58)	3.57(1.93)
Tumor location, No. (%)		
Cardia	3(8.82%)	5(10.42%)
Body	12(35.29%)	19(39.58%)
Pylorus	17(50.00%)	23(47.92%)
Whole stomach	2(5.88%)	1(2.08%)
Macroscopic types, No. (%)		
Borrmann 0	6(17.64%)	12(25.00%)
Borrmann Ⅰ	0(0.00%)	3(6.25%)
Borrmann Ⅱ	2(5.88%)	8(16.67%)
Borrmann Ⅲ	23(67.65%)	22(45.83%)
Borrmann Ⅳ	3(8.82%)	3(6.25%)
Depth of invasion, No. (%)		
Mucosa and submucosa	6(17.65%)	12(25.00%)
Muscularis propria	6(17.65%)	5(10.42%)
Subserosa	13(38.24%)	17(35.42%)
Serosa	5(14.71%)	10(20.83%)
Perforating serosa	4(11.76%)	4(8.33%)
Stage, No. (%)		
Early gastric cancer	6(17.68%)	12(25.00%)
Advanced gastric cancer	28(82.35%)	36(75.00%)
Lymph node metastasis, No. (%)		
Metastasis	16(47.06%)	21(43.75%)
No metastasis	18(52.94%)	27(56.25%)
